# Microencapsulated equine mesenchymal stromal cells promote cutaneous wound healing *in vitro*

**DOI:** 10.1186/s13287-015-0037-x

**Published:** 2015-04-11

**Authors:** Leen Bussche, Rebecca M Harman, Bethany A Syracuse, Eric L Plante, Yen-Chun Lu, Theresa M Curtis, Minglin Ma, Gerlinde R Van de Walle

**Affiliations:** Baker Institute for Animal Health, College of Veterinary Medicine, Cornell University, 235 Hungerford Hill Road, Ithaca, NY 14850 USA; Department of Biological Sciences, State University of New York at Cortland, 21 Graham Avenue, Cortland, NY 13045 USA; Department of Biological and Environmental Engineering, Cornell University, Wing Road, Ithaca, NY 14850 USA

## Abstract

**Introduction:**

The prevalence of impaired cutaneous wound healing is high and treatment is difficult and often ineffective, leading to negative social and economic impacts for our society. Innovative treatments to improve cutaneous wound healing by promoting complete tissue regeneration are therefore urgently needed. Mesenchymal stromal cells (MSCs) have been reported to provide paracrine signals that promote wound healing, but (i) how they exert their effects on target cells is unclear and (ii) a suitable delivery system to supply these MSC-derived secreted factors in a controlled and safe way is unavailable. The present study was designed to provide answers to these questions by using the horse as a translational model. Specifically, we aimed to (i) evaluate the *in vitro* effects of equine MSC-derived conditioned medium (CM), containing all factors secreted by MSCs, on equine dermal fibroblasts, a cell type critical for successful wound healing, and (ii) explore the potential of microencapsulated equine MSCs to deliver CM to wounded cells *in vitro*.

**Methods:**

MSCs were isolated from the peripheral blood of healthy horses. Equine dermal fibroblasts from the NBL-6 (horse dermal fibroblast cell) line were wounded *in vitro*, and cell migration and expression levels of genes involved in wound healing were evaluated after treatment with MSC-CM or NBL-6-CM. These assays were repeated by using the CM collected from MSCs encapsulated in core-shell hydrogel microcapsules.

**Results:**

Our salient findings were that equine MSC-derived CM stimulated the migration of equine dermal fibroblasts and increased their expression level of genes that positively contribute to wound healing. In addition, we found that equine MSCs packaged in core-shell hydrogel microcapsules had similar effects on equine dermal fibroblast migration and gene expression, indicating that microencapsulation of MSCs does not interfere with the release of bioactive factors.

**Conclusions:**

Our results demonstrate that the use of CM from MSCs might be a promising new therapy for impaired cutaneous wounds and that encapsulation may be a suitable way to effectively deliver CM to wounded cells *in vivo*.

**Electronic supplementary material:**

The online version of this article (doi:10.1186/s13287-015-0037-x) contains supplementary material, which is available to authorized users.

## Introduction

Cutaneous wound healing is comprised of a network of biological processes, collectively restoring the integrity of the skin after injury. Unfortunately, the ideal outcome of cutaneous wound healing, which encompasses complete tissue regeneration, is often sacrificed in favor of quickly closing a wound with formation of fibrotic scar tissue [1]. Fibrotic scar formation is an undesirable result of cutaneous wound healing, not only for cosmetic reasons but because scar tissue has compromised mechanical strength and is more sensitive to pain than healthy skin [2]. Treating cutaneous skin wounds and reducing scar tissue cause a financial burden worldwide, and annual expenditures on products designed to minimize scarring exceed $5 billion [3].

To understand the processes involved in cutaneous wound healing or treat fibrotic scar tissue or both, researchers commonly use laboratory rodents as models. Although relevant information has been obtained from studies using rodents, wound healing in these species does not accurately mimic human tissue regeneration [4-7]. For example, mice heal primarily by contraction due to the presence of the panniculus carnosus in their subcutaneous tissues, whereas humans lack this muscle and instead rely on epithelialization to close cutaneous wounds [7]. Owing to these differences, there is a critical need for an animal wound model that closely mimics the natural processes of healing and scar formation in humans. Horses may prove to be an ideal species in which to study cutaneous wound healing since they also depend on epithelial cell activity to heal skin wounds. In addition, horses and humans both suffer from hypergranulation formation, a pathological process defined as an excess of granulation tissue beyond the amount required to replace the tissue deficit. Hypergranulation tissue is referred to as proud flesh in horses and keloid in humans [8]. These common features make the horse an attractive translational model in which to study the underlying pathogenesis of excessive cutaneous wound healing as well as to evaluate the potential of novel treatments.

Mesenchymal stromal cells (MSCs) are multipotent stromal progenitor cells with regenerative properties that are present in a variety of tissues and organs [9]. MSCs actively contribute to regenerative processes, as they are involved in the inflammatory [10], proliferative [11], and remodeling [12] phases of tissue regeneration. Although MSCs were originally reported to contribute to tissue repair by trans-differentiating into the specific cell types needed to restore injured tissue, recent data indicate paracrine signaling as the primary mechanism for the regenerative effects of MSCs [13,14]. Indeed, we recently identified angiogenic stimulating factors in the conditioned medium (CM) of equine MSCs and showed that these factors could induce proliferation and vessel formation of equine endothelial cells *in vitro* [15]. Practically, this implies that the CM obtained from MSC cultures, which contains all factors secreted by MSCs, may be used as a ‘stem cell-free’ therapy in regenerative medicine. This type of therapy offers several advantages over cellular MSC remedies, including the absence of inherent toxicity, no risk for tumor formation of engrafted cells, and no concerns about allograft-associated immune rejection [16,17]. Previous reports have demonstrated that CM obtained from human MSC cultures can improve cutaneous wound healing, although the underlying mechanisms remain unclear [18,19]. The potential of CM obtained from equine MSC cultures to contribute to wound healing has not been studied to date but is essential in order to take advantage of the horse as a physiologically relevant translational model in which to study cutaneous wound healing.

An important aspect to take into consideration when proposing the use of MSC-derived CM in regenerative medicine is the development of a suitable delivery system for these secreted products. Indeed, optimal spatial distribution and continuous release of factors at the site of injury are two key components of MSC treatment that may significantly improve clinical outcome. Cell microencapsulation, which involves immobilization of the cells within a polymeric semi-permeable membrane, provides a supportive microenvironment for the cells in which they can proliferate and release bioactive factors while being shielded from the external environment [20,21]. These microcapsules can be injected at the transplantation bed, localizing the release of therapeutic factors in a controlled way. A recent study by Xu *et al.* describes the potential use of human MSCs encapsulated in biomaterials for the treatment of cutaneous wounds, and their initial findings were that cells ‘packaged’ in a gelatin/poly(ethylene glycol) biomatrix mediated the early resolution of inflammatory events and facilitated the proliferative phases in wound healing [22]. To the best of our knowledge, however, the use of microencapsulated equine MSCs in equine regenerative medicine has not been explored to date.

Therefore, the aims of the present study were (i) to evaluate the *in vitro* effects of equine MSC-derived CM on equine dermal fibroblasts, a cell type critical for successful wound healing, and (ii) to explore the potential of microencapsulated equine MSCs to deliver the critical components of CM to wounded cells *in vitro*. Our salient findings were that equine MSC-derived CM stimulated the migration of equine dermal fibroblasts and increased the expression levels of genes that positively contribute to wound healing in these cells. In addition, we found that equine MSCs packaged in core-shell hydrogel microcapsules have similar effects on equine dermal fibroblast migration and gene expression, suggesting MSC encapsulation may be a suitable way to effectively deliver products secreted by MSCs to wounded cells *in vivo.*

## Methods

### Cells

Equine MSCs were isolated from the peripheral blood of three healthy warmblood mares between 8 and 12 years old, exactly as described previously [15,23]. The blood collection was approved by the Cornell Institutional Animal Care and Use Committee (#2014-0038). Briefly, blood was collected from the vena jugularis of healthy donor horses, and peripheral blood mononuclear cells were isolated by using density gradient centrifugation on 1.080 g/mL Percoll (GE Healthcare, Little Chalfont, Buckinghamshire, UK) and subsequently seeded at a density of 16 × 10^4^ cells/cm^2^ in a T75 flask in culture medium consisting of low-glucose Dulbecco’s modified Eagle’s medium (Invitrogen, part of Thermo Fisher Scientific, Waltham, MA, USA) supplemented with 30% fetal bovine serum (FBS) (Atlanta Biological, Flowery Branch, GA, USA), 10^−7^ M low dexamethasone (Sigma-Aldrich, St. Louis, MO, USA), and 50 μg/mL gentamycin, 1x penicillin-streptomycin, and 2 mM L-glutamine (all from Life Technologies, Grand Island, NY, USA). Cultures were maintained at 37°C with 5% CO_2_. At 70% confluency, cells were removed from flasks by using 0.25% trypsin-EDTA and further cultured in expansion medium, which is identical to the culture medium but without dexamethasone. Equine MSCs were characterized by immunophenotypical protein profiling by using flow cytometry and their potential for trilineage differentiation, exactly as described previously [15]. In essence, equine MSCs were confirmed to be positive for CD29, CD44, CD90, and CD105 and negative for CD45, CD79α, major histocompatibility complex II (MHC II), and a monocyte/macrophage marker. The successful trilineage differentiation of MSCs toward osteoblasts, chondroblasts, and adipocytes was confirmed by using a range of histochemical stains.

The equine dermal fibroblast cell line NBL-6 (American Type Culture Collection, Manassas, VA, USA) was cultured in standard medium consisting of minimal essential medium (Corning) supplemented with 10% FBS (Atlanta Biochemicals) and 1% penicillin/streptomycin (Invitrogen). Cells were maintained at 37°C with 5% CO_2_.

### Generation of conditioned media and pre-treatments

CM was collected from MSCs after 2 days of culture, when cells were 70% confluent. To this end, 6 × 10^5^ MSCs were seeded in a T75 flask in 8 mL of expansion medium. After 48 hours, medium was collected, centrifuged twice for 7 minutes at 300 *g* to remove any cellular debris, and used for further experiments. CM from NBL-6 cells was used as a control and was collected after 2 days of culture, exactly as described for MSC-derived CM. For pre-treatment experiments, MSCs were seeded in expansion medium supplemented with 10 ng/mL tumor necrosis factor-alpha (TNFα) (R&D Systems, Minneapolis, MN, USA), 20 ng/mL interferon-gamma (IFNγ) (R&D Systems), or 150 μM cobalt chloride (CoCl_2_) (Sigma-Aldrich). After 24 hours of culture, cells were washed twice with phosphate-buffered saline (PBS) and fed with 8 mL of fresh expansion medium. CM was collected 24 hours later, as described above.

### Microencapsulated mesenchymal stromal cells

For experiments using encapsulated MSCs, 6 × 10^5^ MSCs were seeded per T75 flask in 8 mL of expansion medium, and in parallel the same number of cells were encapsulated in double-layer microparticles by using a multi-fluidic electrostatic cell micropackaging technique [24]. Briefly, type I collagen neutralized by 1 N sodium hydroxide was mixed with MSCs in expansion medium for a final concentration of 0.45 mg/mL. Cells supported by collagen were encapsulated in 0.9% (wt/vol) alginate hydrogel (FMC Biopolymers, Philadelphia, PA, USA) at a concentration of about 37 cells per capsule. Microencapsulated MSCs were incubated in a crosslinking bath with 100 mM calcium chloride and 5 mM barium chloride under an electrical field strength of 7 kV. Microencapsulated MSCs were resuspended in 8 mL of expansion medium and maintained at 37°C with 5% CO_2_. Empty core-shell hydrogel microcapsules, containing no cells, were included as negative control. After 48 hours, supernatants were collected, centrifuged twice for 7 minutes at 300 *g* to remove cellular debris, and used as CM in experiments.

### *In vitro* scratch assays

NBL-6 cells were seeded in six-well plates at a density of 6 × 10^4^ cells/cm^2^. Upon 90% confluency (after approximately 24 hours), cells were washed twice with PBS and serum-starved overnight. A linear defect was inflicted on the monolayer by using a 200-μL pipette tip. Culture medium was immediately removed (along with any dislodged cells) and replaced with freshly collected CM, diluted 1:1 in expansion medium. Similar scratch assays were repeated in the presence of 2 μg/mL mitomycin C, which was added at the time of scratch infliction. Reference marks were made across the bottoms of the wells with an ultrafine marker. Images of scratches were captured by using a Nikon Diaphot-TMD inverted light microscope with an attached Cohu charge-coupled device (CCD) camera (Nikon, Melville, NY, USA), leaving the reference marks outside the capture image field but within the eye-piece field of view for repeatable orientation of scratches. Photographs of scratches were taken at 0, 24, and 48 hours after scratching, and migration distances of cells were calculated in a blinded-manner by using ImageJ [25]. Widths of scratches were taken in two places in each of three wells scratched per treatment, for a total of six measurements per treatment. Scratch width was subtracted from the time 0 scratch widths at the same location to determine cell migration distance. Migration distances within treatments were averaged to determine overall migration.

### Electric Cell-substrate Impedance Sensing

Cell migration was measured by Electric Cell-substrate Impedance Sensing (ECIS) by using the ECIS Model Z instrument with a 96 W array station (ECIS; Applied BioPhysics Inc., Troy, NY, USA). Dermal fibroblasts were seeded at a density of 2 × 10^5^ cells per well in a 96W1E^+^ PET array chip (Applied BioPhysics Inc.) in standard medium. After 24 hours, medium was removed and replaced with serum-free medium for at least 8 hours prior to wounding. A uniform circular defect was created in the cell monolayer by lethal electroporation (1,200 μA, 40 kHz) for 40 seconds by 350-μm diameter circular electrodes located in each well of the array chip. The defect created in the fibroblast monolayer will be referred to as wound throughout the article, as originally described by Keese *et al.* [26]. Dead cells were detached from the electrode by gently pipetting medium up and down three times before removal. Control CM or MSC-derived CM was added to appropriate wells, and an alternating current (approximately 1 μA, 32 kHz) was applied to the electrodes to measure impedance (in ohms) and monitor wound closure in real time. As cells migrate to repair the circular wound, they physically re-cover the electrode, causing an increase in impedance. The time required for re-covering the electrode was determined as the number of hours it took for the impedance levels in wells with the 350-μm circular wound to return to the same values as the impedance levels in control wells in which cells were not wounded. Cell migration was also documented by phase-contrast images captured by using an Olympus CCX41 inverted microscope equipped with an Olympus LC20 digital camera (Olympus, Tokyo, Japan).

### Gene expression analyses

NBL-6 cells were cultured in 15 × 100 mm culture dishes until confluent. Cells were serum-starved overnight and scratches were made. After two washes with PBS, 1:1 diluted CM derived from MSCs (un-stimulated, stimulated, or encapsulated) or control NBL-6 was added to each NBL-6 culture. After 24 hours, CM was removed, monolayers were rinsed with PBS, and cells were further cultured for another 24 hours. Subsequently, mRNA was extracted from the cells by using an RNEasy Plus Kit in accordance with instructions of the manufacturer (Qiagen, Valencia, CA, USA), followed by a DNase digestion using DNase I (Invitrogen). cDNA was synthesized by using M-MLV Reverse Transcriptase (USB, Cleveland, OH, USA) in accordance with the protocol of the manufacturer. SYBER green-based quantitative polymerase chain reaction (qPCR) assays were carried out on an Applied Biosystems 7500 Fast Real-Time PCR instrument (Applied Biosystems, Carlsbad, CA, USA) to determine fold changes of genes of interest in NBL-6 cells incubated with CM of MSCs compared with NBL-6 incubated with control CM. The comparative threshold cycle (C_t_) method (2^−ΔΔCt^) was used to quantify gene expression levels in which ΔΔC_t_ = ΔC_t_(sample) – ΔC_t_(reference). The previously validated reference gene SDHA (succinate dehydrogenase complex, subunit A) was used to normalize samples [27]. Primers to amplify CCL2, CxCL10, IL-8, MMP1, MMP2, TMP1, plasminogen activator urokinase (PLAU), tissue plasminogen activator (PLAT), PAI1, CTSK, annexin A2 (ANXA2), COLIII, COL1, syndecan 2 (SDC2), SDC4, and fibronectin were designed by using Primer3 software, based on horse sequences found in the National Center of Biotechnology Information GenBank, and primer sequences are listed in Table 1. All samples were run in triplicate.

**Table 1:** **Overview of primers used for semi-quantitative reverse transcription-polymerase chain reaction**

**Gene**	**Abbreviation**	**Forward primer (5′-3′)**	**Reverse primer (5′-3′)**
Succinate dehydrogenase complex, subunit A	*SDHA*	TCCATCGCATAAGAGCAAAG	GGTGGAACTGAACGAACTCC
Plasminogen activator urokinase	*PLAU*	TGTGAGATCACTGGCTTTGG	TGACATTCCTGGTGGGAAAC
Annexin A2	*ANXA2*	GCAGTGTCTGTCACCTCCAG	CCTCCTTCTTGATGCTCTCC
Cathepsin K	*CTSK*	TCCAGAAGGGAAACAAGCAC	TCTTATTCCGAGCCATGAGG
CC chemokine-2	*CCL2*	GGCTCAGCCAGATGCAATTA	GCTTTCTTGTCCAGCTGCTT
Metallopeptidase-2	*MMP2*	GAAGGCTGTGTTCTTTGCAG	TCCAGTTAAAGGCAGCATCC
Syndecan-4	*SDC4*	GCAGCATCTTTGAGAGGACAG	GTTTCTTGCCCAGGTCGTAG
Metallopeptidase-1	*MMP1*	GCCAAATGGACTTCAAGCTG	TAGGAAAGCCGAAGGATCTG
Tissue plasminogen activator	*PLAT*	AGTTCTTGCTGGCTCCTGTC	ATCGGTATGTTCTGCCCAAG
Interleukin-8	*IL-8*	AGACGCACTCCAAACCTTTC	CAGACCTCAGCTCCGTTGAC
Metallopeptidase inhibitor-1	*TIMP1*	GCTCCCTGGAACAGTCTGAG	CGTCCACAAGCAATCAGTGTC
Plasminogen activator inhibitor-1	*PAI1*	CCCTGGAGAGTGAAGTGGAC	CCTGCGATACATGGAGAAGC
Collagen type 1A1	*COL1*	AAGGACAAGAGGCACGTCTG	GCAGGAAAGTCAGCTGGATG
Collagen type 3	*COLIII*	GGGTATAGCTGGTCCTCGTG	GCGCCTCTTTCTCCTTTAGC
Fibronectin	Fibronectin	AGGTCGTTACTGTGGGCAAC	TAATGGGAGACGGTGTAGGG
Syndecan-2	*SDC2*	GACTCAGAAAGGAACGTGGAC	ATAACTCCGCCAGCAATGAC
CXC chemokine-10	*CxCL10*	GACTCTGAGTGGAACTCAAGGAAT	GTGGCAATGATCTCAACACG

### Cell viability assays

To ensure that mitomycin C was not toxic to NBL-6 fibroblasts, cells were seeded at 10,000 per well in 96-well microplates. At 90% confluency, mitomycin C was added to triplicate wells at 0, 2, 20, 200, and 2,000 ng/mL. After 48 hours of culture, an MTT (3-(4,5-dimethylthiazol-2-yl)-2,5-diphenyltetrazolium bromide) *in vitro* toxicology assay (Sigma-Aldrich) was carried out in accordance with the instructions of the manufacturer and absorbance was measured at 570 nm on a Multiskan EX plate reader (Thermo Fisher Scientific). Optical densities of wells treated with mitomycin C were compared with those of untreated wells to determine cell viability.

To test the viability of microencapsulated MSCs, capsules were dissolved on days 2, 7, 12, 17, and 22 after encapsulation by using a solution of 1% EDTA. The released MSCs were washed with culture medium, incubated for 3 minutes with trypsin-EDTA to create a single cell suspension, and washed with culture medium, and the percentage of living cells was determined by using the Trypan blue exclusion (TBE) assay.

### Population doubling time calculations

For determination of population doubling time (PDT), 2 × 10^5^ released MSCs were plated in a T25 tissue culture flask and cultured in expansion medium. The PDT was calculated by using the following link: [28]. PDTs of microencapsulated MSCs were compared with PDTs of non-encapsulated MSCs from the same horse at the same passages.

### Characterization of mesenchymal stromal cells after encapsulation

MSCs removed from capsules 2 days after encapsulation were plated in T75 tissue culture flasks containing expansion medium until monolayer was 90% confluent. At that time, cells were collected for flow cytometric analyses and seeded for differentiation assays as described previously [15].

### Proliferation assays

The effect of mitomycin C on the proliferation of NBL-6 was evaluated by using a bromodeoxyuridine (BrdU) proliferation assay kit (Abcam, Cambridge, MA, USA). To this end, NBL-6 were seeded on a 96-well plate at a density of 5 × 10^4^ cells per well and incubated with mitomycin C for 48 hours. Regular medium was used to establish baseline proliferation. The BrdU proliferation assay kit was used in accordance with the instructions of the manufacturer, and the resulting absorbance was read at 450 nm on a Multiskan EX microplate reader by using Ascent software (Thermo Scientific, Waltham, MA, USA). Empty wells and wells without BrdU were included as controls.

### Statistical analyses

Student’s *t* test for paired data was used to test for statistically significant differences in cell migration (scratch assays and ECIS), mRNA expression (RT-qPCR), cell viability (TBE and MTT), and BrdU incorporation (enzyme-linked immunosorbent assay, or ELISA) between untreated and CM-stimulated NBL-6. Data given are the mean of three replicates, and the bars show standard deviations (SDs). To compare data from different individuals, statistical analysis was done by means of the non-parametric Kruskal-Wallis and Dunn’s mulitiple comparison test (α = 0.05), and significant differences were considered at *P* value of less than 0.01.

## Results

### Conditioned medium from mesenchymal stromal cells stimulates migration of dermal fibroblasts in scratch assays

**Figure 1:**
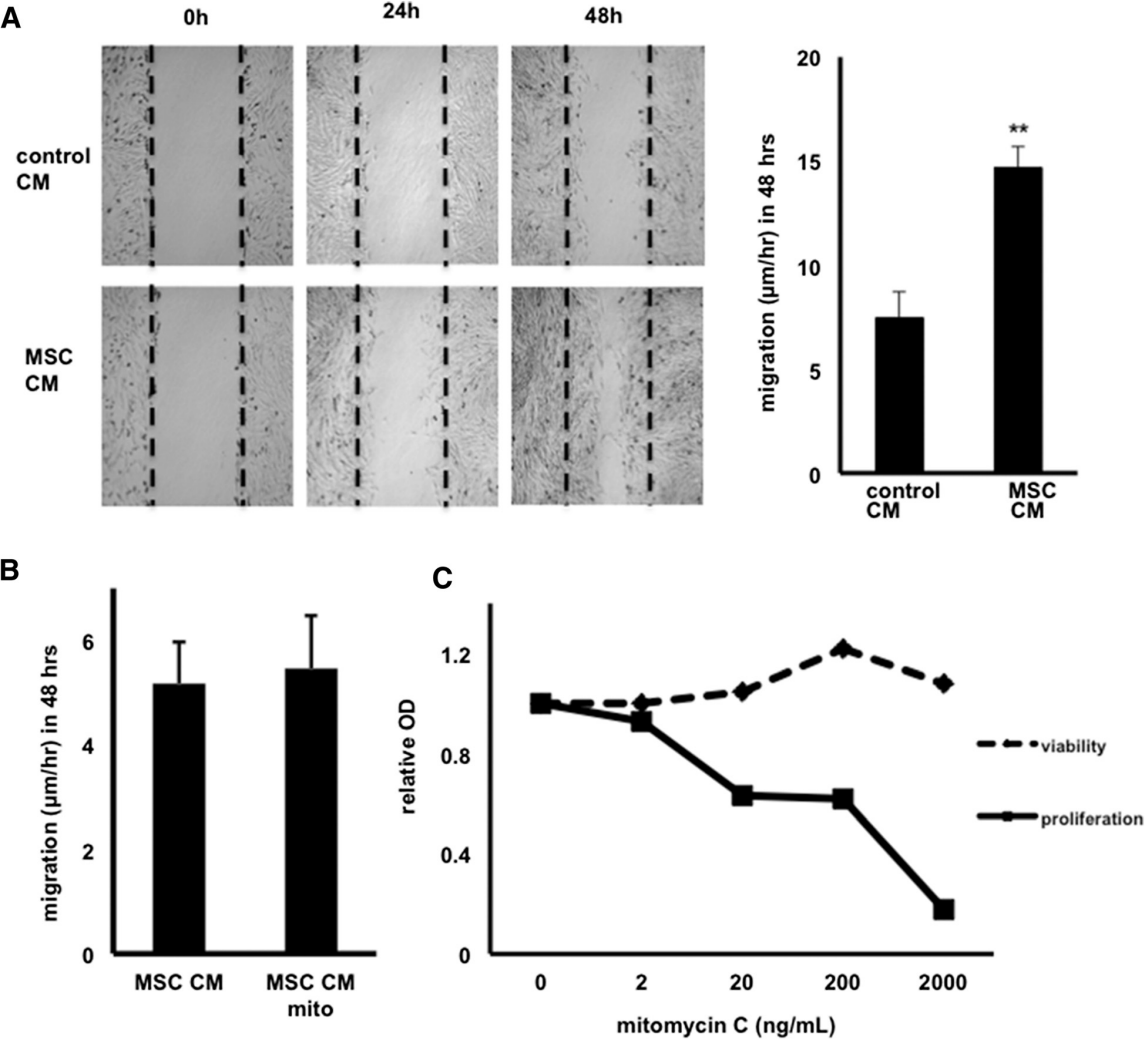
Mesenchymal stromal cell (MSC) conditioned medium (CM) promotes migration of dermal fibroblasts in scratch assays (n = 3). **(A)** Representative phase-contrast images of wounded NBL-6 cells cultured with MSC-derived CM (lower panels) as compared with control CM (upper panels) over a 48-hour period, and migration distances of NBL-6 cells are expressed as micrometer per hour in 48 hours. **P <0.01. **(B)** Migration of NBL-6 cells cultured with MSC-derived CM as compared with CM from mitomycin C-treated MSCs (2,000 ng/mL). **(C)** Viability (dashed line) and proliferation (solid line) of NBL-6 cells cultured in the presence of various concentrations of mitomycin C. NBL-6, horse dermal fibroblast cell; OD, optical density.

Because dermal fibroblasts are critically involved in wound healing [29], we decided to study the effects of MSC-derived CM on the migration of these cells by using *in vitro* scratch assays [30]. We found that equine dermal fibroblast cells (NBL-6) migrated significantly faster when cultured in CM obtained from equine MSCs compared with control CM, which was collected from NBL-6 (Figure 1A).

Given the high incidence of hypergranulation formation in equine and human wounds [8], it was important to explore whether this increased rate of wound closure was due to enhanced migration or increased proliferation of the fibroblasts or both. Therefore, we repeated the scratch assays in the presence of mitomycin, an inhibitor of proliferation. No differences in migration of NBL-6 cells cultured in MSC-derived CM in the presence or absence of mitomycin were found, indicating that the observed effect in the scratch assays was not due to an increased proliferation of the cells (Figure 1B). To ensure that the mitomycin concentration used for these experiments (that is, 2,000 ng/mL) was effective in inhibiting proliferation without affecting cell viability, we performed a BrdU ELISA as well as an MMT assay, respectively (Figure 1C).

**Figure 2:**
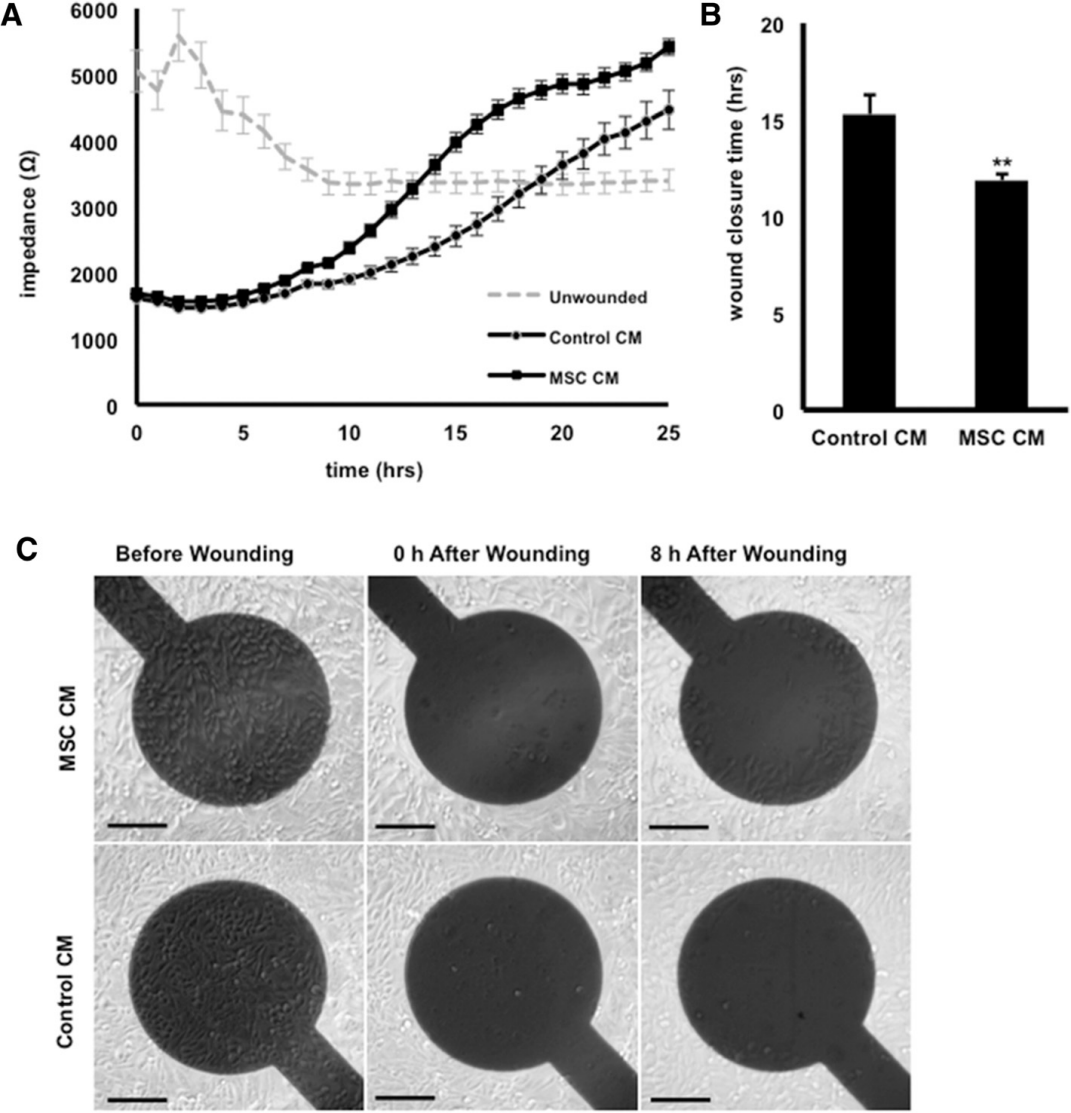
Mesenchymal stromal cell (MSC) conditioned medium (CM) promotes wound closure using Electric Cell-substrate Impedance Sensing (n = 3). Wound-healing rates of NBL-6 cells cultured with MSC-derived CM or control CM, as determined by electrical impedance in ohms detected from 0 to 24 hours after wounding **(A)** and total wound closure time expressed in hours **(B)**. **P <0.01. **(C)** Phase-contrast images of wounded NBL-6 cells cultured with MSC-derived CM (upper panels) as compared with control CM (lower panels) over a 24-hour period. Scale bars = 100 μm. NBL-6, horse dermal fibroblast cell.

To corroborate our findings on the increased migration of fibroblasts when exposed to MSC-derived CM, ECIS experiments were used to measure *in vitro* wound closure rates [31]. Again, we found that equine dermal fibroblasts migrated significantly faster when cultured in CM obtained from equine MSCs compared with control CM with a wound closure of 11.8 ± 0.35 hours versus 15.3 ± 0.96 hours, respectively (Figure 2A, B). Because the ECIS system creates only a very small wound (350 μm) that normally heals in 12 to 18 hours, cell migration rather than proliferation is the major contributor to closure under these conditions (Figure 2C). Interestingly, the ending impedance value shown in Figure 2A of the MSC CM-treated fibroblasts was higher than that of the control, indicating that the addition of the MSC CM may increase either cell-cell or cell-matrix contacts on the healed fibroblast monolayer leading to a higher final impedance.

### Conditioned medium of mesenchymal stromal cells induces alterations in the expression of genes involved in wound healing

**Figure 3:**
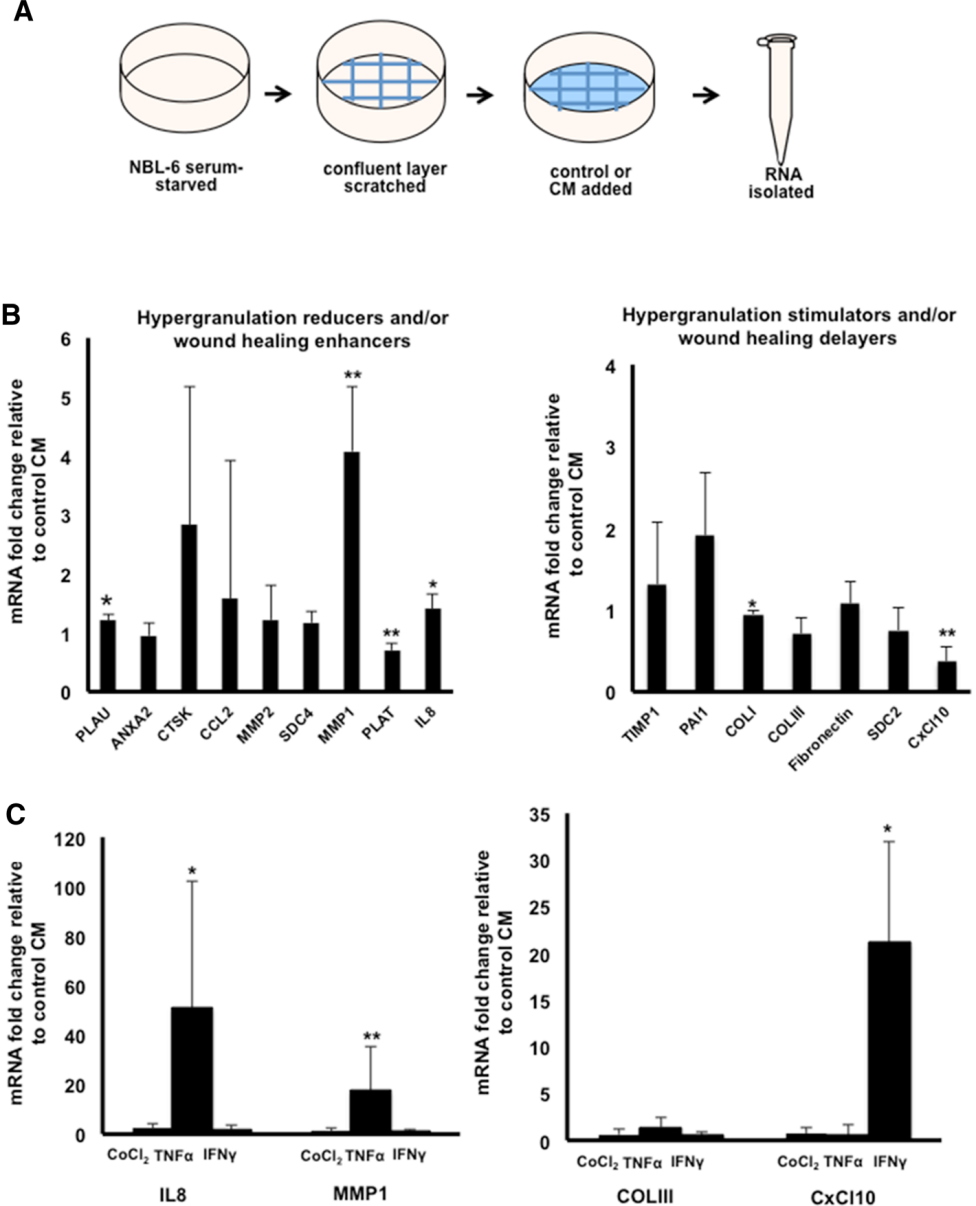
Mesenchymal stromal cell (MSC) conditioned medium (CM) alters gene expression levels in dermal fibroblasts (n = 3). **(A)** Schematic illustration of NBL-6 treatments for RNA isolation. Fold change of mRNA levels, as detected by quantitative reverse transcription-polymerase chain reaction, in **(B)** NBL-6 cells cultured with MSC-derived CM as compared with control CM or **(C)** NBL-6 cells cultured with CM from preconditioned MSCs as compared with CM from non-preconditioned MSCs. Left panel shows genes involved in reduction of hypergranulation or enhancement of wound healing or both, and right panel shows genes that stimulate hypergranulation or delay wound healing or both. *P <0.05; **P <0.01. ANXA2, annexin A2; CCL2, CC chemokine 2; CoCl_2_, cobalt chloride; COLI, collagen type 1A1; COLIII, collagen type 3; CTSK, cathepsin K; CxCL10, CXC chemokine 10; IFNγ, interferon-gamma; IL-8, interleukin-8; MMP1, metallopeptidase 1; MMP2, metallopeptidase 2; NBL-6, horse dermal fibroblast cell; PAI1, plasminogen activator inhibitor-1; PLAT, tissue plasminogen activator; PLAU, plasminogen activator urokinase; SDC2, syndecan 2; SDC4, syndecan 4; TIMP1, metallopeptidase inhibitor 1; TNFα, tumor necrosis factor-alpha.

Semi-quantitative RT-PCR analyses were performed to investigate the relative mRNA expression levels of selected inflammatory cytokines, remodeling enzymes, extracellular matrix components, and adhesion molecules, all associated with wound repair, in NBL-6 cultured with MSC-derived CM as opposed to control CM. Figure 3A shows the experimental set-up for these studies, and it was observed that culturing dermal fibroblasts in the CM of MSCs differentially regulated mRNA expression of several genes involved in wound healing when compared with gene expression in dermal fibroblasts cultured in control CM (Figure 3B). A significant upregulation of the pro-inflammatory mediator interleukin-8 (IL-8) (*P* <0.05) and a significant downregulation of the chemokine C-X-C motif chemokine 10 (CxCL10) (*P* <0.01) were observed in dermal fibroblasts cultured in the presence of MSC-derived CM (Figure 3B). Gene expression levels of extracellular matrix components and adhesion molecules were not significantly different, with the exception of collagenase 3 (COLIII), which was significantly lower in dermal fibroblasts cultured in the presence of MSC-derived CM compared with control CM (*P* <0.05) (Figure 3B). Finally, the expression of a variety of remodeling enzymes was upregulated when dermal fibroblasts were cultured in CM, with matrix metalloprotease protein 1 (MMP1) showing a significantly higher expression in dermal fibroblasts cultured in the presence of MSC-derived CM compared with control CM (*P* <0.01) (Figure 3B).

### Conditioned medium of mesenchymal stromal cells stimulated with TNFα, IFNγ, or COCl_2_ further alters gene expression in dermal fibroblast cells but does not result in an increased migration capacity

Since it has been reported previously that pre-stimulation of human MSCs increased their wound-healing properties [32-35], we stimulated our equine MSCs with TNFα, IFNγ, and COCl_2_ to evaluate the effects on (i) expression of wound healing-related genes in NBL-6 cells and (ii) *in vitro* scratch assays. It was observed that stimulation of MSCs with TNFα led to a significant increase in MMP1 (*P* <0.01) and IL-8 (*P* <0.05) mRNA expression in NBL-6 cells but that stimulation with IFNγ led to a significant increase in CxCL10 expression (*P* <0.05) compared with culturing NBL-6 cells with CM of non-stimulated MSCs (Figure 3C). In contrast, none of the stimulating agents influenced the expression of COLIII, and stimulation of MSCs with CoCl_2_ had no effect on the expression of any of the evaluated genes (Figure 3C). During evaluation of these pre-stimulation treatments in scratch assays, no differences in migration rates were observed between fibroblasts exposed to the stimulated MSC-derived CM versus the non-stimulated MSC-derived CM (Additional file 1: Figure S1A, B).

### Effects of mesenchymal stromal cell-derived conditioned medium on the expression of wound healing-related genes are donor-dependent in our study

When we studied the changes in gene expression in more detail, it became clear that potential significant differences in gene expression levels could be masked because of the rather large SDs we observed for several tested genes. Since we used the CM of MSCs from three different individual horses for our experiments, we reasoned that these large SDs could be caused by natural inter-horse variations. Therefore, we decided to present the results of the gene expression alterations per individual donor horse instead of using the average (Additional file 2: Figure S2A). By presenting the data this way, we made several observations. Firstly, when the non-parametric Kruskal-Wallis test was used to compare the gene expression data between horses, statistically significant differences between individuals became apparent in the mRNA expression levels of certain genes (Table 2). At a *P* value of less than 0.01, there were statistically significant differences for two hypergranulation reducers or wound-healing enhancers or both, namely cathepsin K (CTSK) and matrix metalloproteinase-2 (MMP2) (Table 2A). Likewise, statistically significant differences (*P* <0.01) between horses were found for four hypergranulation enhancers or wound-healing reducers or both, namely plasminogen activator inhibitor 1 (PAI1), collagenase type III (COLIII), fibronectin, and SDC2 (Table 2B). Secondly, with Dunn’s *post hoc* test, it was observed that culturing NBL-6 cells in the MSC-derived CM of horse B resulted in a significant mean rank difference of at least 6 for the genes CTSK, MMP2, PAI1, and COLIII when compared with NBL-6 cells that were cultured in the CM of either horse A- or horse C-derived MSCs (Additional file 2: Figure S2B). Overall, the increased expression levels of CTSK and MMP2, genes advantageous for wound healing, with a simultaneously decreased expression level of PAI1, a gene disadvantageous for wound healing, would make horse B a better donor for providing MSCs with the capacity to promote wound healing than either horse A or C. Using scratch assays to compare the effects of horse B MSC-derived MSCs on migration of fibroblasts with the other two horse MSC samples revealed no statistically significant differences and all three MSC samples could equally increase NBL-6 migration (*P* <0.01, Additional file 2: Figure S2C). Taken together, these data indicate that the expression level pattern in CM-treated dermal fibroblast cells is donor horse MSC-specific in our study.

**Table 2:** **Statistical analyses of gene expression data from individual horses**

**Gene**	**Significant difference**		
	**Horse A versus horse B**	**Horse B versus horse C**	**Horse A versus horse C**
**A. Hypergranulation reducers or wound-healing enhancers or both**			
*PLAU*	-	-	-
*ANXA2*	-	Yes^a^	-
*CTSK*	-	Yes^b^	-
*CCL2*	-	Yes^a^	-
*MMP2*	-	Yes^b^	-
*SDC4*	-	-	-
*MMP1*	-	-	-
*PLAT*	-	-	-
*IL-8*	-	-	Yes^a^
**B. Hypergranulation enhancers or wound-healing reducers or both**			
*TIMP1*	-	Yes^a^	-
*PAI1*	-	Yes^b^	-
*COLI*	-	-	-
*COLIII*	-	Yes^b^	-
Fibronectin	Yes**	-	-
*SDC2*	-	-	Yes^b^

^a^ *P* <0.05; ^b^ *P* <0.01.

### Encapsulated mesenchymal stromal cells retain stem cell characteristics and remain viable during long-term encapsulation

**Figure 4:**
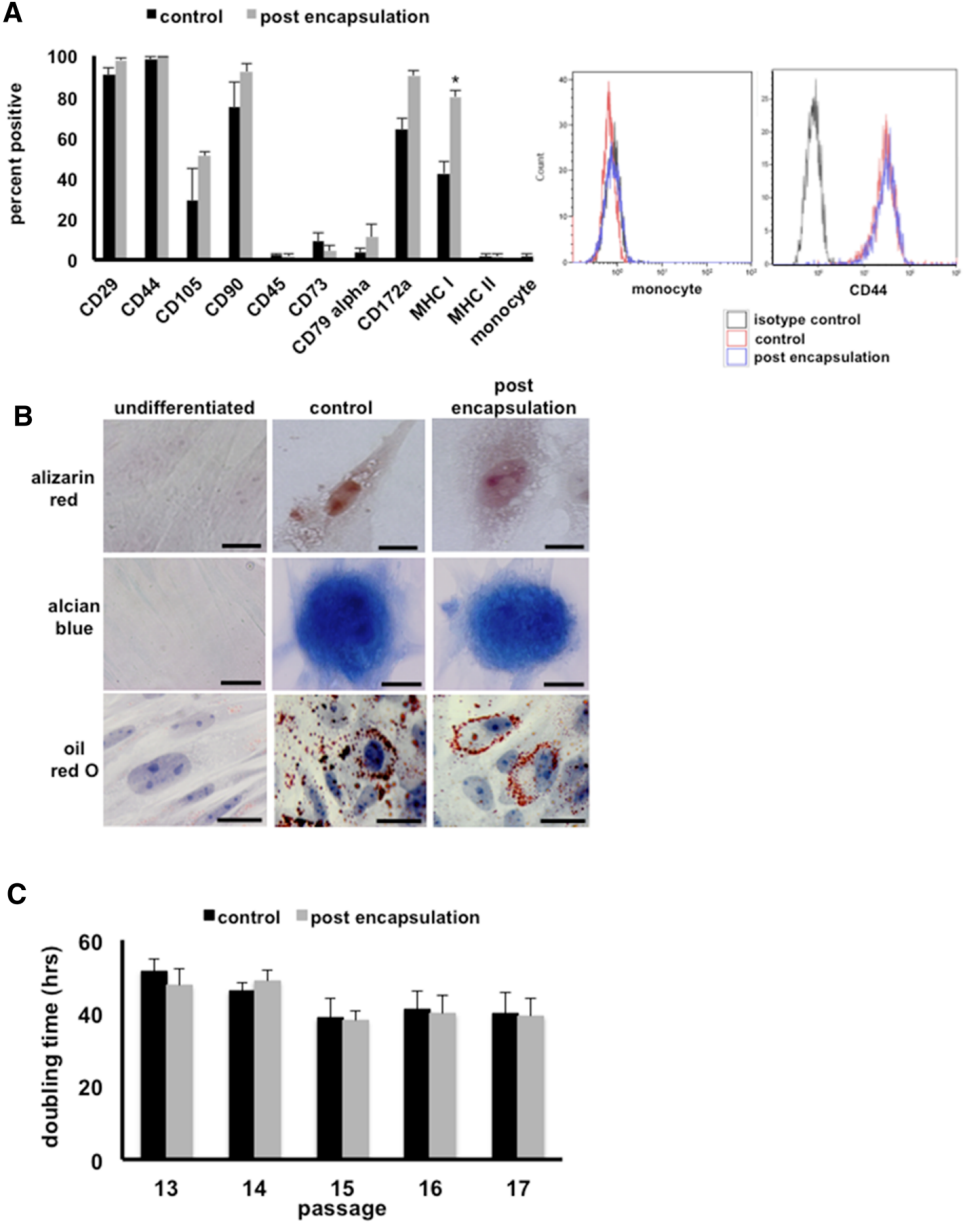
Mesenchymal stromal cells (MSCs) retain stem cell characteristics after microencapsulation (n = 3). **(A)** Expression of surface markers, as detected by flow cytometry, on MSCs removed from capsules after 2 days post-encapsulation as compared with control MSCs that were never encapsulated. Percentage of total cells positive is shown on the left, and representative histograms are shown on the right. *P <0.05. **(B)** Representative images of MSCs stained with Alizarin Red, Alcian Blue, and Oil Red O to detect osteocyte, chondrocyte, and adipocyte differentiation, respectively. Left column: undifferentiated MSCs cultured in expansion medium; center column: control MSCs that were never encapsulated cultured in MSC differentiation media; and right column: MSCs removed from capsules after 2 days post-encapsulation cultured in MSC differentiation media. Scale bars = 10 μm. **(C)** Population doubling times of MSCs removed from capsules after 2 days post-encapsulation as compared with control MSCs that were never encapsulated. MHC, major histocompatibility complex.

After clearly demonstrating that the CM of equine MSCs promotes wound healing, we were interested in exploring the potential of microencapsulating MSCs as a way to deliver the critical components of CM to wounded cells *in vitro* in a controlled manner. In a first set of experiments, we encapsulated equine MSCs and 2 days later removed these MSCs from their capsules to evaluate whether they retained their stem cell characteristics. Overall, the immunophenotypical profile, as detected by flow cytometric analysis using a commonly used set of cell surface markers [36], did not differ between MSCs removed from capsules and MSCs that were never encapsulated (Figure 4A). There was a significant difference (*P* <0.05) in the expression of MHC I, but this marker is known to be highly variably expressed by MSCs [37]. In addition, MSCs removed from capsules retained the capacity to differentiate into osteocyte, chondrocyte, and adipocyte lineages (Figure 4B). Moreover, the PDT of MSCs removed from capsules was virtually identical to that of MSCs from the same source, but never encapsulated, from passage 13 to 17 (end of experiment) (Figure 4C).

**Figure 5:**
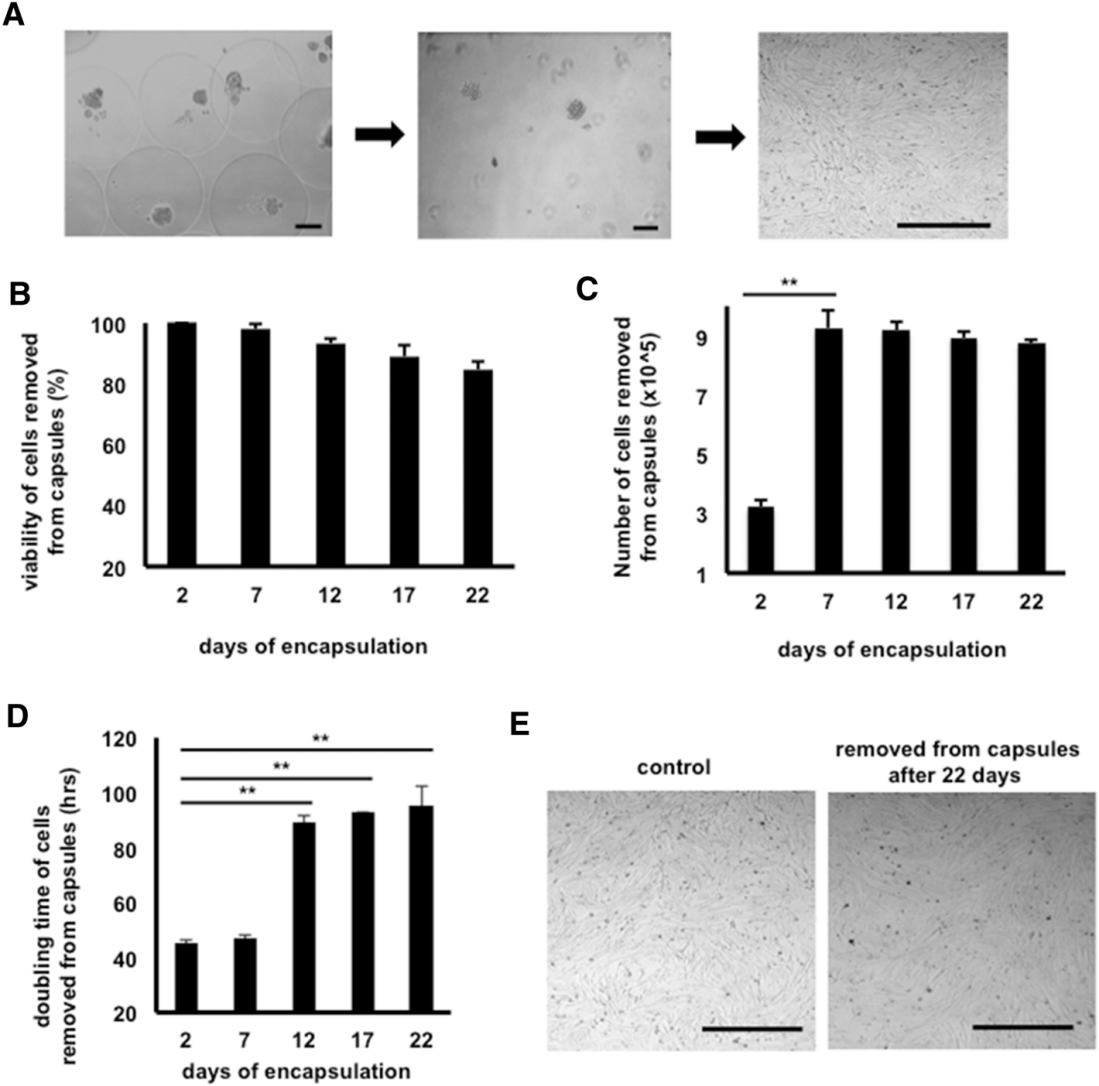
Mesenchymal stromal cells (MSCs) remain viable after long-term microencapsulation (n = 3). **(A)** Representative images of MSCs encapsulated (left), immediately after removal of capsules (center), and after several days of culture (right). **(B)** Viability, as determined by Trypan blue exclusion, of MSCs removed from capsules at 5-day intervals starting at 2 days after encapsulation. **(C)** Numbers of viable cells removed from equal numbers of capsules at 5-day intervals starting at 2 days after encapsulation. **P <0.01. **(D)** Population doubling times of MSCs removed from capsules at 5-day intervals starting at 2 days post-encapsulation. **P <0.01. **(E)** Representative phase-contrast images of cultured MSCs removed from capsules 22 days post-encapsulation as compared with control MSCs that were never encapsulated. Scale bars = 50 μm.

In a second set of experiments, we cultured MSCs in capsules for over 3 weeks in order to test the potential of long-term encapsulation. MSCs encapsulated for 2 days were used as the short-term encapsulation control time point (day 2) and compared with MSCs encapsulated for successive 5-day intervals (days 7, 12, 17, and 22). At all of these different time points, equine MSCs were removed from equal numbers of capsules and subsequently plated in culture wells (Figure 5A). Before plating, viability was determined by using TBE and the number of live cells was determined. Over the course of the experiment, the viability of long-term encapsulated cells decreased slightly compared with short-term encapsulation but did not fall below 80% and this slight decrease was not statistically significant (Figure 5B). In addition, the number of viable cells present in the capsules for 7 days was significantly increased compared with 2-day encapsulation, indicating that equine MSCs did divide in the capsules, but remained constant thereafter (Figure 5C). After plating and upon confluency of the cells, PDTs were calculated. Cells encapsulated for 7 days showed a PDT similar to that of the short-term, 2-day, encapsulated MSCs. In contrast, cells encapsulated for more than 7 days showed a significantly higher PDT, indicating that it took those cells much longer to form a confluent monolayer (Figure 5D). For the duration of this set of experiments, pictures from confluent monolayers were taken daily to confirm that the stem cell morphology was maintained, indicating that the change in PDT was not due to differentiation of the MSCs which could result in an altered growth pattern (Figure 5E).

### Encapsulated mesenchymal stromal cell-derived conditioned medium stimulates migration of dermal fibroblasts and induces alterations in the expression of genes involved in wound healing

After showing that microencapsulated MSCs remain viable and retain general stem cell characteristics, we evaluated the CM from encapsulated MSCs in order to determine potential positive effects on dermal fibroblast migration and expression of genes involved in wound healing. To this end, we repeated the scratch assays and gene expression analyses, as previously described, by using CM from non-encapsulated MSCs (control) and from MSCs that were encapsulated and cultured for 2 days.

**Figure 6:**
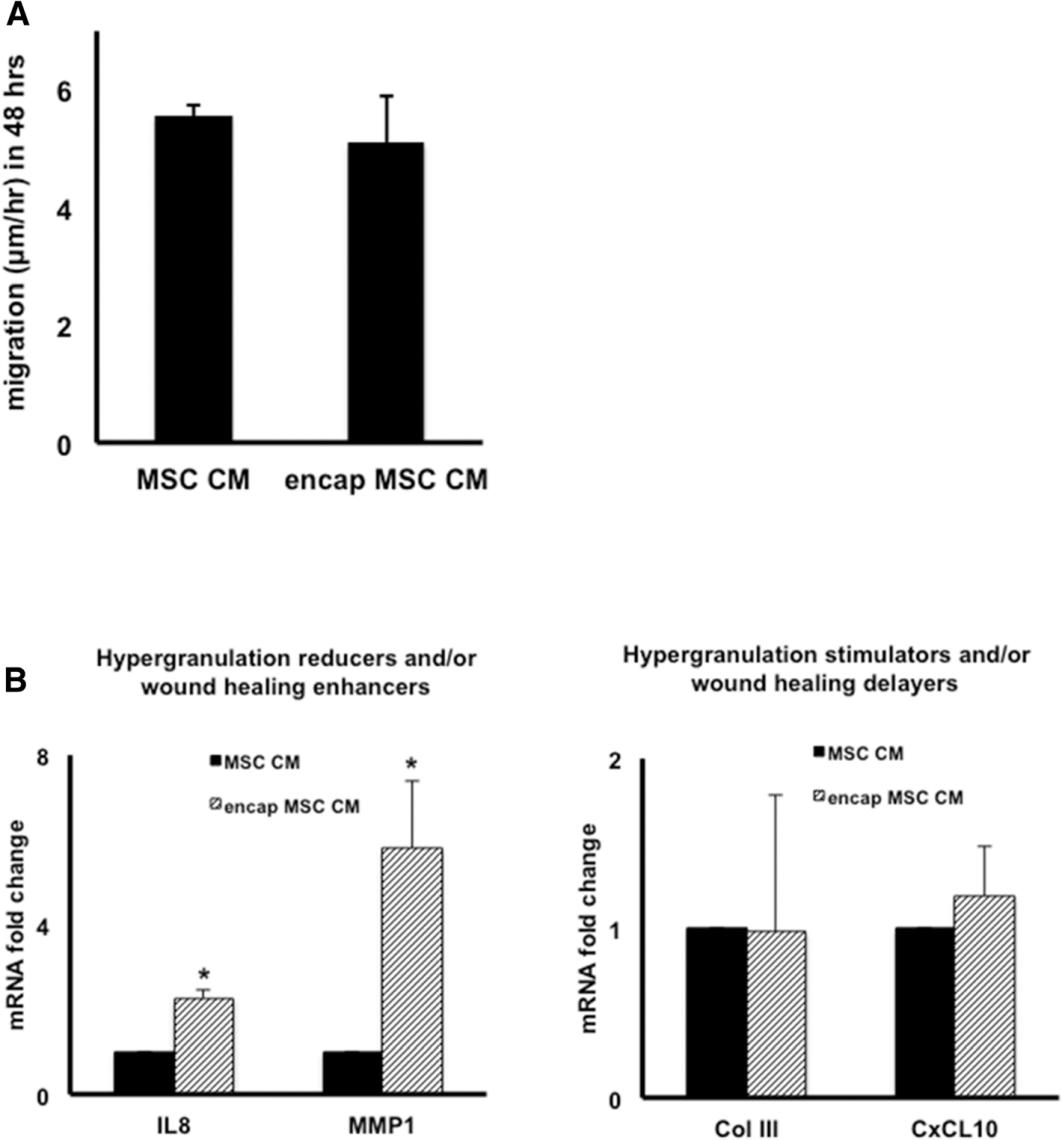
Conditioned medium (CM) from microencapsulated mesenchymal stromal cells (MSCs) promotes NBL-6 migration and alters gene expression (n = 3). **(A)** Migration distances of NBL-6 cells cultured in CM from encapsulated MSCs as compared with MSC CM in *in vitro* scratch assays. Data are expressed as micrometer per hour in 48 hours. **(B)** Fold change of mRNA, as detected by quantitative reverse transcription-polymerase chain reaction, in NBL-6 cells cultured with encapsulated MSC CM as compared with control MSC CM. Left panel shows genes involved in reduction of hypergranulation or enhancement of wound healing or both, and right panel shows genes that stimulate hypergranulation or delay wound healing or both. *P <0.05. COLIII, collagen type 3; CxCL10, CXC chemokine 10; IL, interleukin; MMP1, metallopeptidase 1; NBL-6, horse dermal fibroblast cell.

Using scratch assays, we found that NBL-6 cells had an equally fast migration when cultured in CM obtained from equine MSCs compared with CM obtained from encapsulated equine MSCs (Figure 6A). CM that was collected from 2-day cultured empty capsules was also included as a control and showed a significantly slower migration compared with either MSC-derived CM (data not shown). Next, semi-quantitative RT-PCR analyses were performed on NBL-6 cells cultured in CM obtained from non-encapsulated MSCs (control, set as 1) and compared with gene expression in NBL-6 exposed to CM obtained from encapsulated MSCs. Specifically, the relative mRNA expression levels of a selected set of genes (that is, IL-8, MMP1, ColIII, and CxCL10) involved in wound healing were determined. A significant upregulation of IL-8 and MMP1 gene expression was observed in NBL-6 cells cultured in CM obtained from encapsulated equine MSCs as compared with NBL-6 cells which were cultured in CM of non-encapsulated MSCs (Figure 6B). In contrast, the gene expression levels of COLIII and CxCL10 were indistinguishable between NBL-6 cells cultured in CM from either encapsulated MSCs or non-encapsulated MSCs (Figure 6B).

## Discussion

The present study is the first to evaluate the effects of CM from equine MSCs on wound-healing properties of equine dermal fibroblasts and to explore the potential of encapsulated equine MSCs to deliver these critical components to equine target cells. Our salient findings were that the CM of equine MSCs stimulates the migration of equine dermal fibroblasts *in vitro* and increases the expression levels of genes that positively contribute to wound healing. Importantly, similar results were obtained by using CM from MSCs that were encapsulated in core-shell hydrogel microcapsules, indicating the potential of microencapsulated MSCs to deliver the bioactive factors present in CM.

During our studies on the expression levels of genes involved in wound healing when equine fibroblasts were exposed to MSC-derived CM, two interesting observations were made. First, we found a highly significant, four-fold, increase in the mRNA expression of the MMP1. MMP1, also designated interstitial collagenase or fibroblast collagenase, is a critical remodeling enzyme required for re-epithelialization during wound healing [38,39]. *In vitro* studies have shown that CM from amnion-derived human MSCs alters the expression of MMP1 in human dermal fibroblasts [35], which is similar to our present findings with CM from peripheral blood-derived equine MSCs and equine dermal fibroblasts. Interestingly, the CM collected from MSCs that were microencapsulated even further altered the expression of MMP1 as evidenced by a significantly higher MMP1 expression in dermal fibroblasts when exposed to the CM from microencapsulated MSCs compared with CM from non-encapsulated MSCs (Figure 6B). A similar observation was made for IL-8 expression in dermal fibroblasts (Figure 6B), another critical component in cutaneous wound healing known to stimulate migration and proliferation of keratinocytes [40]. Although it is speculative at this point, we hypothesize that the close contact between the MSCs in these capsules or the capsule microenvironment or both changes the secretion pattern of MSCs or, alternatively, that the MSC secretome is influenced by adherent (non-encapsulated) versus non-adherent (encapsulated) culture conditions. An increased expression level of MMP1 and IL-8 in dermal fibroblasts was also observed when equine MSCs were preconditioned by using TNFα. TNFα has been described to stimulate human MSC to secrete elevated levels of IL6 and IL8, resulting in accelerated wound healing [29]. Our study shows that IL8 is also upregulated in equine target cells exposed to CM from TNFα treated equine MSC. For the two other agents that we used to precondition equine MSCs in our present study, however, we did not observe an upregulation of IL-8 or MMP1 in dermal fibroblasts when exposed to CM from these stimulated MSCs. The first agent, CoCl_2_, is a chemical known to mimic hypoxia, and our results in our equine model reflect results from a previous study in which no significant alterations were found in the expression levels of MMP1 in human dermal fibroblasts incubated with CM from hypoxic human MSCs when compared with CM from normoxic human MSCs [35]. In addition, no significant alterations were found in any of the other genes that were tested (that is, IL-8, COLIII, and CxCL10) when equine fibroblasts were exposed to the CM of CoCl_2_-treated equine MSCs. The second agent we used to stimulate equine MSCs was IFNγ, a cytokine known to stimulate MSCs to increase wound healing in aged mice [33] but for which the specific effects on the expression of wound-related factors in target cells, like dermal fibroblasts, have not previously been investigated. We found in our present study that equine dermal fibroblasts showed a significant increase in CxCL10 expression when incubated with CM of IFNγ-preconditioned MSCs. CxCL10 is a chemokine known to delay wound healing and to disorganize neovascularization [41], and although more in-depth studies are needed, we would like to argue that pretreating MSCs with IFNγ might not be beneficial as a regenerative treatment to promote cutaneous wound healing.

Secondly, we observed that the expression level pattern in CM-treated dermal fibroblast cells was donor horse MSC-specific. Although our current study is too limited in experimental numbers and is lacking repeated isolations from the same donor horse to draw definite conclusions about donor dependency, our data are the first to indicate a donor-specific effect of MSC-derived CM on skin target cells specifically. This study and other studies describing donor variation in the cytokine expression level and *in vitro* bone tissue repair potency of human MSCs [42,43] collectively suggest that screening of candidate MSC donors is critical in order to take full advantage of the therapeutic effects of MSCs on cutaneous wound healing. Moreover, it is important to take into account the specific purpose of the regenerative therapy, as the CM of one donor MSC culture might prove more beneficial than the CM of another MSC culture, depending on the desired therapeutic effect.

Despite the exciting potential of the MSC-derived CM as an effective cell-free therapeutic treatment in regenerative medicine, a major impediment to its use is the fact that these MSC formulations are not fully optimized in terms of delivery methods. The ability to control secretion of MSC-derived bioactive factors is critical given the limitations of pharmacokinetics and stability of proteins *in vivo*. To begin exploring the potential of such delivery methods, we microencapsulated our equine MSCs by using core-shell hydrogel microcapsules. Conventional alginate microcapsules have been used successfully to encapsulate human MSCs [44-47]. However, our study demonstrated that this approach is feasible for equine MSCs by using core-shell capsules which were designed to provide a more relevant extracellular environment and to better protect the encapsulated cells [24]. Indeed, we showed that equine MSCs survive and divide within these microcapsules and retain their stem cell characteristics. Importantly, we found that CM collected from these encapsulated equine MSCs equally promotes dermal fibroblast migration when compared with the CM from non-encapsulated MSCs, suggesting that encapsulating of MSCs may be an ideal strategy to control the delivery of secreted products to equine wounds *in vivo.* Based on data we have collected thus far, future experiments are planned in which we will evaluate the healing-promoting effects of equine MSCs, non-encapsulated as well as microencapsulated, in an *in vivo* horse model, such as the horse model established by the group of Theoret *et al*. in which skin wounds are experimentally induced to heal both normally as well as with the formation of exuberant granulation tissue [48].

## Conclusions

MSCs have been reported to provide paracrine signals that promote wound healing, but (i) how they exert their effects on target cells is unclear and (ii) a suitable delivery system to supply these MSC-derived secreted factors in a controlled and safe way is unavailable. The present study was designed to provide answers to these questions by using the horse as a translational model. Our results suggest that CM of MSCs might be a promising new therapy for impaired cutaneous wounds and that microencapsulation may be a suitable way to effectively deliver CM to wounded cells *in vivo*. Moreover, our data showed that the effects of MSC-derived CM appear to be donor-specific, suggesting that a proper screening of candidate MSC donors is critical in order to take full advantage of the therapeutic effects of MSCs on cutaneous wound healing.

## Additional files

Additional file 1: Figure S1. Conditioned medium (CM) from preconditioned mesenchymal stromal cells (MSCs) does not affect migration of dermal fibroblasts (n = 3). **(A)** Representative phase-contrast images of wounded NBL-6 cells cultured with CM of MSCs stimulated with tumor necrosis factor-alpha (TNFα), interferon-gamma (IFNγ), or cobalt chloride (CoCl_2_) as compared with control MSC CM at 0, 24, and 48 hours. **(B)** Migration distances of NBL-6 cells cultured with CM of MSCs stimulated with TNFα, IFNγ, or CoCl_2_ as compared with control MSC CM. Data are expressed as micrometers per hour in 48 hours. NBL-6, horse dermal fibroblast cell. Additional file 2: Figure S2. Mesenchymal stromal cell (MSC) conditioned medium (CM) alters horse dermal fibroblast cell (NBL-6) gene expression in a donor-specific manner (n = 3). **(A)** Fold change of mRNA, as detected by quantitative reverse transcription-polymerase chain reaction (qRT-PCR), in NBL-6 cells cultured with MSC CM from three different donor horses as compared with control NBL-6 CM. Left panel shows genes involved in reduction of hypergranulation or enhancement of wound healing or both, and right panel shows genes that stimulate hypergranulation or delay wound healing or both. **(B)** Expression levels of cathepsin K (CTSK) and metallopeptidase 2 (MMP2) (genes involved in reduction of hypergranulation or enhancement of wound healing or both) and plasminogen activator inhibitor-1 (PAI1) and collagen type 3 (ColIII) (genes that stimulate hypergranulation or delay wound healing or both) with a significant mean rank difference of at least 6 upon incubation with horse B MSC-derived CM. **(C)** Migration distances of NBL-6 cells cultured with CM of MSCs from three different donor horses as compared with control CM. Data are expressed as micrometers per hour in 48 hours. ***P* <0.01.

## Abbreviations

BrdU: bromodeoxyuridine
CCL2: CC chemokine 2
CM: conditioned medium
CoCl_2_: cobalt chloride
COLI: collagen type 1A1
COLIII: collagen type 3
Ct: threshold cycle
CTSK: cathepsin K
CxCL10: CXC chemokine 10
ECIS: Electric Cell-substrate Impedance Sensing
ELISA: enzyme-linked immunosorbent assay
FBS: fetal bovine serum
IFNγ: interferon-gamma
IL: interleukin
MHC: major histocompatibility complex
MMP: metallopeptidase
MSC: mesenchymal stromal cell
MTT: 3-(4,5-dimethylthiazol-2-yl)-2,5-diphenyltetrazolium bromide
NBL-6: horse dermal fibroblast cell
PAI1: plasminogen activator inhibitor-1
PBS: phosphate-buffered saline
PDT: population doubling time
qPCR: quantitative polymerase chain reaction
RT-PCR: reverse transcription-polymerase chain reaction
SD: standard deviation
SDC: syndecan
TBE: Trypan blue exclusion
TIMP1: metallopeptidase inhibitor 1
TNFα: tumor necrosis factor-alpha
