# Supplementary figures and images for: Microencapsulated equine mesenchymal stromal cells promote cutaneous wound healing in vitro
Source: Stem Cell Res Ther. 2015 Apr 11;6(1):66. doi: 10.1186/s13287-015-0037-x (PMC4413990; doi:10.1186/s13287-015-0037-x)

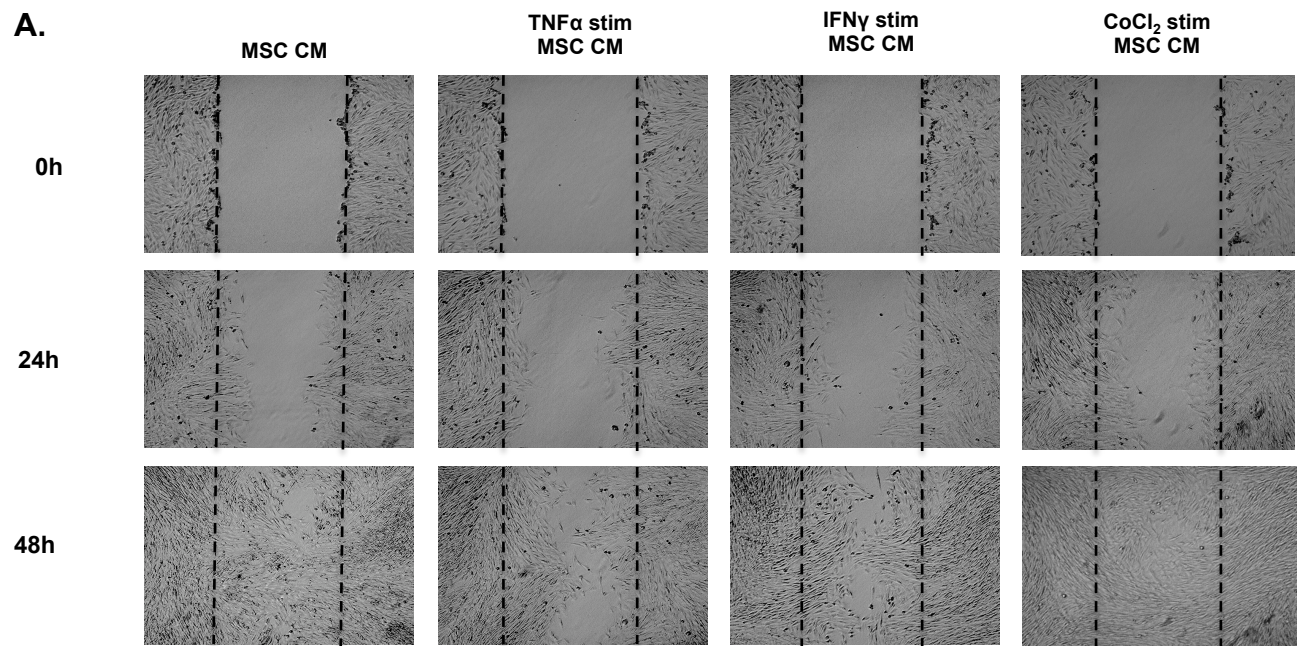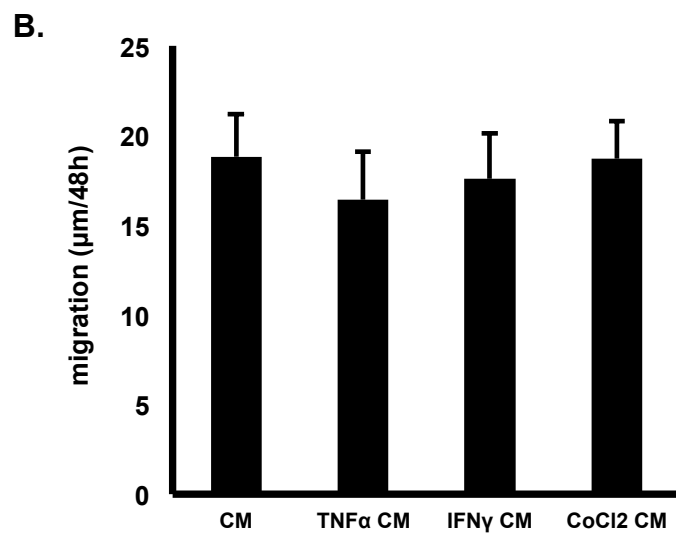

**Supplemental Figure 1. Bussche et al.**

Supplement: Additional file 1: Figure S1. — Conditioned medium (CM) from preconditioned mesenchymal stromal cells (MSCs) does not affect migration of dermal fibroblasts (n = 3). (A) Representative phase-contrast images of wounded NBL-6 cells cultured with CM of MSCs stimulated with tumor necrosis factor-alpha (TNFα), interferon-gamma (IFNγ), or cobalt chloride (CoCl2) as compared with control MSC CM at 0, 24, and 48 hours. (B) Migration distances of NBL-6 cells cultured with CM of MSCs stimulated with TNFα, IFNγ, or CoCl2 as compared with control MSC CM. Data are expressed as micrometers per hour in 48 hours. NBL-6, horse dermal fibroblast cell. [file 13287_2015_37_MOESM1_ESM.pdf]

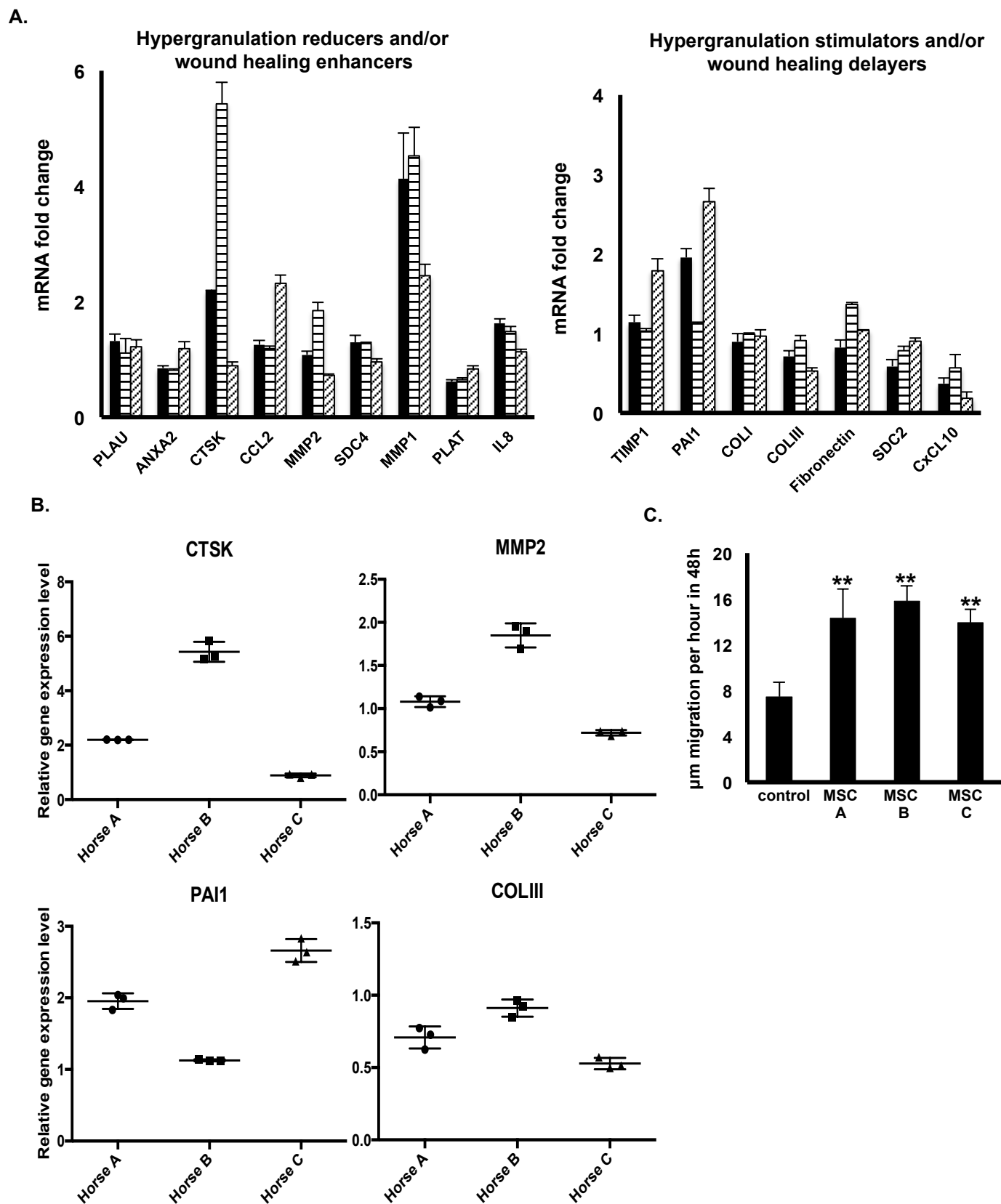

Supplemental Figure 2. Bussche et al.

Supplement: Additional file 2: Figure S2. — Mesenchymal stromal cell (MSC) conditioned medium (CM) alters horse dermal fibroblast cell (NBL-6) gene expression in a donor-specific manner (n = 3). (A) Fold change of mRNA, as detected by quantitative reverse transcription-polymerase chain reaction (qRT-PCR), in NBL-6 cells cultured with MSC CM from three different donor horses as compared with control NBL-6 CM. Left panel shows genes involved in reduction of hypergranulation or enhancement of wound healing or both, and right panel shows genes that stimulate hypergranulation or delay wound healing or both. (B) Expression levels of cathepsin K (CTSK) and metallopeptidase 2 (MMP2) (genes involved in reduction of hypergranulation or enhancement of wound healing or both) and plasminogen activator inhibitor-1 (PAI1) and collagen type 3 (ColIII) (genes that stimulate hypergranulation or delay wound healing or both) with a significant mean rank difference of at least 6 upon incubation with horse B MSC-derived CM. (C) Migration distances of NBL-6 cells cultured with CM of MSCs from three different donor horses as compared with control CM. Data are expressed as micrometers per hour in 48 hours. **P <0.01. [file 13287_2015_37_MOESM2_ESM.pdf]
